# Losses of lifetime employment duration and productivity for patients with different subtypes and stages of lung cancer

**DOI:** 10.1007/s10198-023-01624-4

**Published:** 2023-08-07

**Authors:** Szu-Chun Yang, Wu-Wei Lai, Tzu-I. Wu, Jing-Shiang Hwang, Jung-Der Wang, Fuhmei Wang

**Affiliations:** 1 Department of Internal Medicine, College of Medicine, National Cheng Kung University Hospital, National Cheng Kung University, Tainan, Taiwan; 2 Department of Surgery, College of Medicine, National Cheng Kung University Hospital, National Cheng Kung University, Tainan, Taiwan; 3 Department of Public Health, College of Medicine, National Cheng Kung University, Tainan, Taiwan; 4 Institute of Statistical Science, Academia Sinica, Taipei, Taiwan; 5 Department of Occupational and Environmental Medicine, College of Medicine, National Cheng Kung University Hospital, National Cheng Kung University, Tainan, Taiwan; 6 Department of Economics, College of Social Sciences, National Cheng Kung University, No.1 University Road, Tainan, 701 Taiwan

**Keywords:** Productivity, Employment, Human capital, Lung cancer, LDCT screening

## Abstract

**Background:**

How different subtypes and stages of lung cancer affect morbidity- and mortality-associated productivity have not been investigated. This study quantified the losses of lifetime employment duration and productivity among patients with various subtypes and stages of lung cancer.

**Methods:**

We identified nationwide lung cancer patients diagnosed at the ages of 50–64 between 2011 and 2019. Monthly survival probabilities were weighted by monthly employed-to-population ratios and working salaries to estimate lifetime employment duration and productivity. We compared lifetime employment duration and productivity of patients with those of the age-, sex-, calendar year-matched general population for losses of lifetime employment duration and productivity, which were multiplied by pathology and stage shifts based on the first-round screening of Taiwan Lung Cancer Screening in Never Smoker Trial (TALENT) to calculate the savings of lifetime employment duration and productivity.

**Results:**

Lung cancer patients had shorter survival and employment duration than the referents. Patients with lung cancers other than adenocarcinoma experienced greater losses of lifetime employment duration and productivity as compared to adenocarcinoma patients. Applying the estimations of never-smoking patients to 100 lung cancer patients with pathology and stage shifts based on the TALENT, the savings of lifetime employment duration and productivity were 132.2 (95% prediction interval: 116.2–147.4) years and 3353 (95% prediction interval: 2914–3802) thousand US dollars, respectively.

**Conclusions:**

Early diagnosis of lung cancer would save the losses of employment duration and lifetime productivity. Future evaluation of the cost-effectiveness of lung cancer screening could consider incorporating these societal impacts.

**Supplementary Information:**

The online version contains supplementary material available at 10.1007/s10198-023-01624-4.

## Introduction

Lung cancer is the leading cause of cancer death worldwide [1]. Screening with low-dose computed tomography (LDCT) has changed the outlook of lung cancer management. Due to the premature mortality, lung cancer patients experience the greatest loss of productivity among all cancer patients [2–4]. Notably, lung cancer patients aged 50 to 64 years, or the age range eligible for LDCT screening, experience the largest degree of productivity loss [5]. Among all cancer survivors, lung cancer patients have the highest decline in employment rates [6–8]. Approximately half of lung cancer survivors lose their jobs after treatment [9]. Mortality and morbidity-related productivity loss not only diminishes the quality of life for lung cancer patients [10] but also places a financial burden on their family and society.

Several previous investigations have estimated the years of productive life lost and the cost of productivity loss for lung cancer patients [3–5]. However, these investigations only calculated the productivity loss related to premature mortality. Although some studies have evaluated morbidity-associated productivity loss [11, 12], no study has analyzed the productivity loss stratified by pathological subtypes and tumor stages. Advanced stage is a major predictor of unemployment and indirect costs for lung cancer patients [11, 13]. Estimating the productivity loss according to different lung cancer subtypes and stages may help evaluate the benefits of early diagnosis.

The first-round screening of Taiwan Lung Cancer Screening in Never Smoker Trial (TALENT) showed a high detection rate of early-stage lung cancer [14]. However, the societal impact of this screening strategy has not been investigated. This study aimed to quantify the losses of lifetime employment duration and productivity for patients with different pathological subtypes and stages of lung cancer, particularly those who were never smokers. These estimates could be multiplied by the pathology and stage shifts resulted from the TALENT screening to estimate the savings of lifetime employment duration and productivity.

## Materials and methods

### Data source

We linked Taiwan National Cancer Registry database (2011–2019) with the National Mortality Registry (2011–2020) and the National Health Insurance (NHI) reimbursement (2011–2020) databases for analysis. These databases encompass 99% of the nation’s inhabitants and are representative of the Taiwanese population [15]. As the eligible participants for lung cancer screening are between 50 and 80 years old [16], we identified lung cancer patients diagnosed at ages 50–64 as the index cohort. The reference cohort consisted of age-, sex-, and calendar year-matched individuals from the general population in Taiwan. To calculate the losses of lifetime employment duration and productivity. We compared the life expectancy (LE), lifetime employment duration, and lifetime productivity of index patients with those of the referents. Subgroup analysis was conducted for never-smoking patients, and the results were multiplied by pathology and stage shifts based on the first-round screening of TALENT [14] to estimate the savings of lifetime employment duration and productivity. This study was approved by the Institutional Review Board of National Cheng Kung University Hospital (A-ER-110-287), and informed consent was waived due to the use of de-identified information.

### Lifetime survival

We identified pathologically-verified lung cancer patients newly diagnosed at ages 50–64 from the National Cancer Registry database using the International Classification of Diseases for Oncology, third edition (ICD-O-3) codes C34.0-C34.9. Lung cancer was stratified by histological subtypes, including small cell lung cancer (SCLC), adenocarcinoma, non-small cell lung cancers (NSCLCs) other than adenocarcinoma. Tumor stage was defined according to the American Joint Committee on Cancer classifications. We linked patient identification information to the National Mortality Registry database and followed up until the end of 2020. We applied the Kaplan–Meier method to estimate the survival function, and extrapolated the survival to lifetime using the following steps: First, we simulated survival of the age- and sex-matched referents by using the life tables in the patients’ onset years, and estimated the Kaplan–Meier survival function of the reference population. Second, we calculated the survival ratio between the index cohort and reference population at each time point *t* and performed logit transformation of the survival ratio. Third, a restricted cubic splines model was applied to fit the logit-transformed survival ratio, which was almost linear. The logit-transformed survival ratio from the fitted restricted cubic splines model and the survival function of the reference population were used to extrapolate the index patients’ survival to the next month. The above one-month survival extrapolation procedures were repeatedly implemented to obtain the lifetime survival function of the index cohort. Loss of LE was the difference between the LE of lung cancer patients and that of the age-, sex-, calendar year-matched referents. Detailed methods regarding survival extrapolation have been demonstrated [17] and validated in lung cancer patients [18, 19].

We used the survival data of patients who were diagnosed during the first 5 years (2011–2015) and extrapolated them to 10 years (2011–2020). Because these patients were actually followed from 2011 until the end of 2020, the mean survival duration within the 10-year follow-up period using Kaplan–Meier method was considered as the gold standard. The estimates using the extrapolation based on the first 5 years of follow-up were compared with the 10-year follow-up Kaplan–Meier estimates to obtain the relative biases.

### Loss of lifetime employment duration

We identified monthly employment status of the index patients and their corresponding referents to capture the dynamic changes in employment rates over time. The NHI database contains employment information on each beneficiary as follows: employer, employed in a public sector, employed in a private sector, and unemployed. Monthly employed-to-population ratio (EMRATIO) was defined as the ratio of individuals employed at that month to the total number of people in the corresponding age- and sex-specific strata. The EMRATIOs of individuals aged ≥ 65 were assumed to be 0 in this study. Because some index patients were not followed up until age 65 or death, we extrapolated the EMRATIO using the following equation:

$${\text{logit }}\left( {E\left( {t|i} \right)} \right){-}{\text{logit }}(E(t|r))\, = \,\beta _{0} \, + \,\beta _{1} \left( {{\text{log }}\left( {h\left( {t|i} \right)} \right){-}{\text{log }}\left( {h\left( {t|r} \right)} \right)} \right)\, + \,\varepsilon _{t}$$

where *E*(*t*|*i*) and *E*(*t*|*r*) denote the EMRATIOs at time point *t* for index patients and corresponding referents, respectively, and *h*(*t*|*i*) and *h*(*t*|*r*) denote the mortality hazards at time point *t* for index patients and corresponding referents, respectively. That is, we assumed that EMRATIO decreases over time as the mortality risk increases and a linear relationship exists between the difference in logit-transformed EMRATIOs and difference in log-transformed hazards*.* A linear regression model was applied to fit the difference in logit-transformed values to extrapolate the EMRATIOs of the index patients beyond the follow-up limit. To avoid the influence of outliers due to the small sample size at the end of follow-up, monthly EMRATIOs of index patients with a sample size of < 50 were excluded [20].

We multiplied the survival functions by the monthly EMRATIOs of index patients and matched referents to estimate their respective lifetime employment durations. Loss of lifetime employment duration was defined as the difference between the lifetime employment duration of lung cancer patients and that of the age-, sex-, calendar year-matched referents [21]. Relative loss of lifetime employment duration associated with lung cancer was calculated as the loss of lifetime employment duration divided by the lifetime employment duration of the referents.

### Loss of lifetime productivity

In addition to the differences in monthly employment, the monthly working salaries of index patients were expected to differ from those of referents. According to prior research, the working salary inferred from one’s NHI insurance premium was 90.2% (employees in public sectors) to 113.3% (employees in private sectors) of the working salary reported in the National Family Income and Expenditure Survey [22]. We multiplied the survival functions by the monthly working salaries of index patients and referents to estimate their respective lifetime productivities. Loss of lifetime productivity was the difference between the lifetime productivity of lung cancer patients and that of referents. Loss of lifetime productivity divided by the lifetime productivity of referents was relative loss of lifetime productivity associated with lung cancer.

The 95% confidence intervals for the estimates of LE, loss of lifetime employment duration, and loss of lifetime productivity were obtained through 100 bootstraps.

### Savings of lifetime employment duration and productivity

We performed a subgroup analysis of lung cancer patients who were never smokers. Taiwan Lung Cancer Screening in Never Smoker Trial (TALENT) is a single-arm study that provided pathology and stage information for lung cancer cases diagnosed during the first-round screening [14]. We compared the distributions of pathology and stage between the national never-smoker subgroup with lung cancer patients diagnosed at ages 50–64 in the TALENT. The losses of lifetime employment duration and productivity for lung cancer patients with specific pathology and stage were multiplied by the pathology and stage shifts to estimate savings of lifetime employment duration and productivity. We simulated 100 patients diagnosed as lung cancer with different Dirichlet distributions of pathology and stage, and performed cohort simulation with 1000 iterations to obtain the 95% prediction intervals.

## Results

A total of 33,118 patients were diagnosed with lung cancer between the ages of 50 and 64 from 2011 to 2019. Table 1 summarizes the demographic and clinical characteristics of the index patients by pathology and stage. Most patients with SCLC or NSCLC other than adenocarcinoma were male, while more than half of adenocarcinoma patients were female. Adenocarcinoma patients were generally younger and more likely to be employed, particularly those with stage IA tumors. The majority of lung cancer patients worked in the private sector. The interquartile ranges of the monthly working salaries used for calculating the insurance premiums ranged from US$459 and US$1527. Patients with stage IA adenocarcinoma paid the highest premiums. Most patients with SCLC or NSCLC other than adenocarcinoma were ever smokers, while the majority of adenocarcinoma patients were never smokers. SCLC or non-adenocarcinoma patients were more likely to have a Charlson Comorbidity Index score of ≥ 3 compared to adenocarcinoma patients.

**Table 1:** Characteristics of lung cancer patients in Taiwan from 2011 to 2019 (*N* = 33,118) by pathology and stage

	SCLC		Adenocarcinoma					Non-Adeno			
	Limited	Extensive	IA	IB	II	III	IV	I	II	III	IV
Number	761	1831	5423	1564	776	2129	13,848	753	510	1946	3577
* Age, n (%)*											
50–54	142 (18.7)	376 (20.5)	1499 (27.7)	349 (22.3)	176 (22.7)	474 (22.3)	3280 (23.7)	152 (20.2)	108 (21.2)	441 (22.7)	816 (22.8)
55–59	261 (34.3)	573 (31.3)	1845 (34.0)	503 (32.2)	257 (33.1)	737 (34.6)	4802 (34.7)	232 (30.8)	176 (34.5)	645 (33.1)	1165 (32.6)
60–64	358 (47.0)	882 (48.2)	2079 (38.3)	712 (45.5)	343 (44.2)	918 (43.1)	5766 (41.6)	369 (49.0)	226 (44.3)	860 (44.2)	1596 (44.6)
Male,* n (%)*	680 (89.4)	1614 (88.2)	1793 (33.1)	665 (42.5)	364 (46.9)	1153 (54.2)	6845 (49.4)	564 (74.9)	425 (83.3)	1617 (83.1)	2743 (76.7)
*Classification of employment, n (%)*											
Employer	25 (3.3)	52 (2.8)	274 (5.0)	68 (4.3)	34 (4.4)	87 (4.1)	453 (3.3)	29 (3.9)	12 (2.4)	57 (2.9)	103 (2.9)
Public sector	24 (3.1)	61 (3.3)	470 (8.7)	106 (6.8)	42 (5.4)	100 (4.7)	614 (4.4)	39 (5.2)	23 (4.5)	74 (3.8)	108 (3.0)
Private sector	379 (49.8)	813 (44.4)	2821 (52.0)	779 (49.8)	360 (46.4)	1068 (50.2)	6781 (49.0)	378 (50.2)	290 (56.8)	977 (50.2)	1656 (46.3)
Unemployed	333 (43.8)	905 (49.5)	1858 (34.3)	611 (39.1)	340 (43.8)	874 (41.0)	6000 (43.3)	307 (40.7)	185 (36.3)	838 (43.1)	1710 (47.8)
*Monthly salary for the insurance premium,mean (IQR) US$*	852 (670–1160)	812 (596–1160)	1353 (733–1527)	1129 (730–1463)	1035 (670–1463)	1003 (670–1337)	939 (670–1273)	962 (730–1273)	935 (730–1210)	852 (642–1160)	802 (459–1060)
*Smoking status, n (%)*											
Never smokers	77 (10.1)	219 (12.0)	4281 (78.9)	1076 (68.8)	462 (59.5)	1073 (50.4)	7883 (56.9)	231 (30.7)	112 (22.0)	405 (20.8)	1047 (29.3)
Ever smokers	635 (83.5)	1477 (80.7)	1026 (18.9)	417 (26.7)	257 (33.1)	906 (42.6)	4928 (35.6)	461 (61.2)	358 (70.2)	1357 (69.7)	2084 (58.2)
Unknown	49 (6.4)	135 (7.3)	116 (2.2)	71 (4.5)	57 (7.4)	150 (7.0)	1037 (7.5)	61 (8.1)	40 (7.8)	184 (9.5)	446 (12.5)
*CCI score, n (%)*											
0	277 (36.4)	724 (39.5)	2036 (37.5)	640 (40.9)	288 (37.1)	863 (40.5)	6428 (46.4)	234 (31.1)	159 (31.2)	644 (33.1)	1439 (40.2)
1	163 (21.4)	384 (21.0)	1102 (20.3)	320 (20.5)	178 (22.9)	466 (21.9)	2846 (20.5)	160 (21.3)	103 (20.2)	447 (23.0)	700 (19.6)
2	133 (17.5)	264 (14.4)	1121 (20.7)	303 (19.4)	156 (20.1)	376 (17.7)	1809 (13.1)	126 (16.7)	103 (20.2)	322 (16.5)	517 (14.4)
≥ 3	188 (24.7)	459 (25.1)	1164 (21.5)	301 (19.2)	154 (19.9)	424 (19.9)	2765 (20.0)	233 (30.9)	145 (28.4)	533 (27.4)	921 (25.8)

*CCI* Charlson Comorbidity Index, *IQR* interquartile range, *Non-adeno* non-small cell lung cancers other than adenocarcinoma, *SCLC* small cell lung cancer

Among the 17,605 subcohort diagnosed in the first 5 years (2011–2015), survival curves were extrapolated to 2020 and compared with the Kaplan–Meier estimates based on the actual 10-year follow-up (Supplementary Table 1). The relative biases of the extrapolation ranged from -6.4% and 5.7%.

### Losses of lifetime employment duration and productivity

**Fig. 1:**
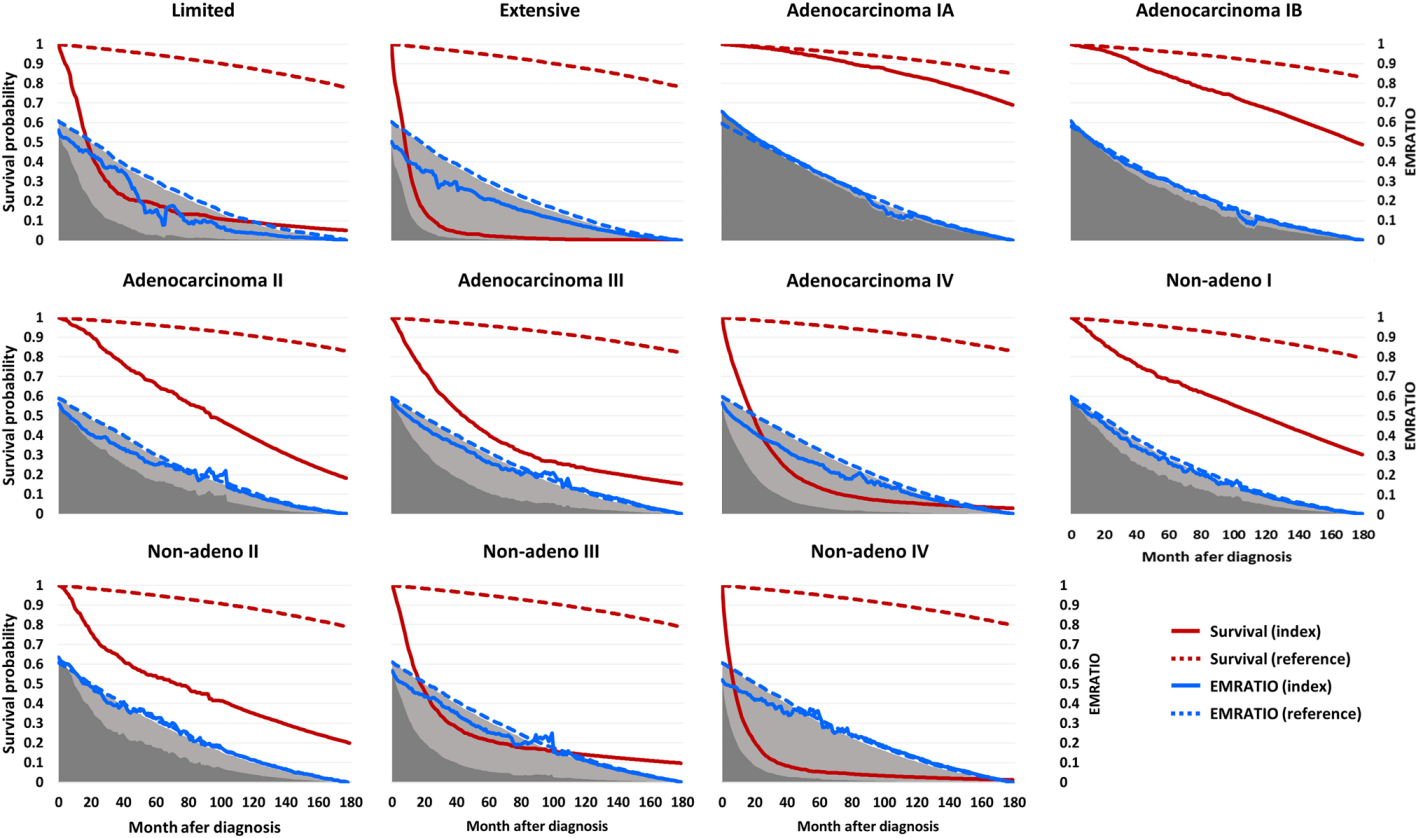
Lifetime employment durations (dark shaded areas) and losses of employment duration (light shaded areas) of lung cancer patients. The latter depict the differences in lifetime employment duration between the index and age-, sex-, and calendar year-matched reference groups. The reddish curves represent the survival probabilities along time after diagnosis; the blue curves indicate the employed-to-population ratios (EMRATIOs); among them, the index groups were shown as solid lines, while the corresponding reference groups were expressed as dashed lines. Non-adeno: non-small cell lung cancers other than adenocarcinoma

**Fig. 2:**
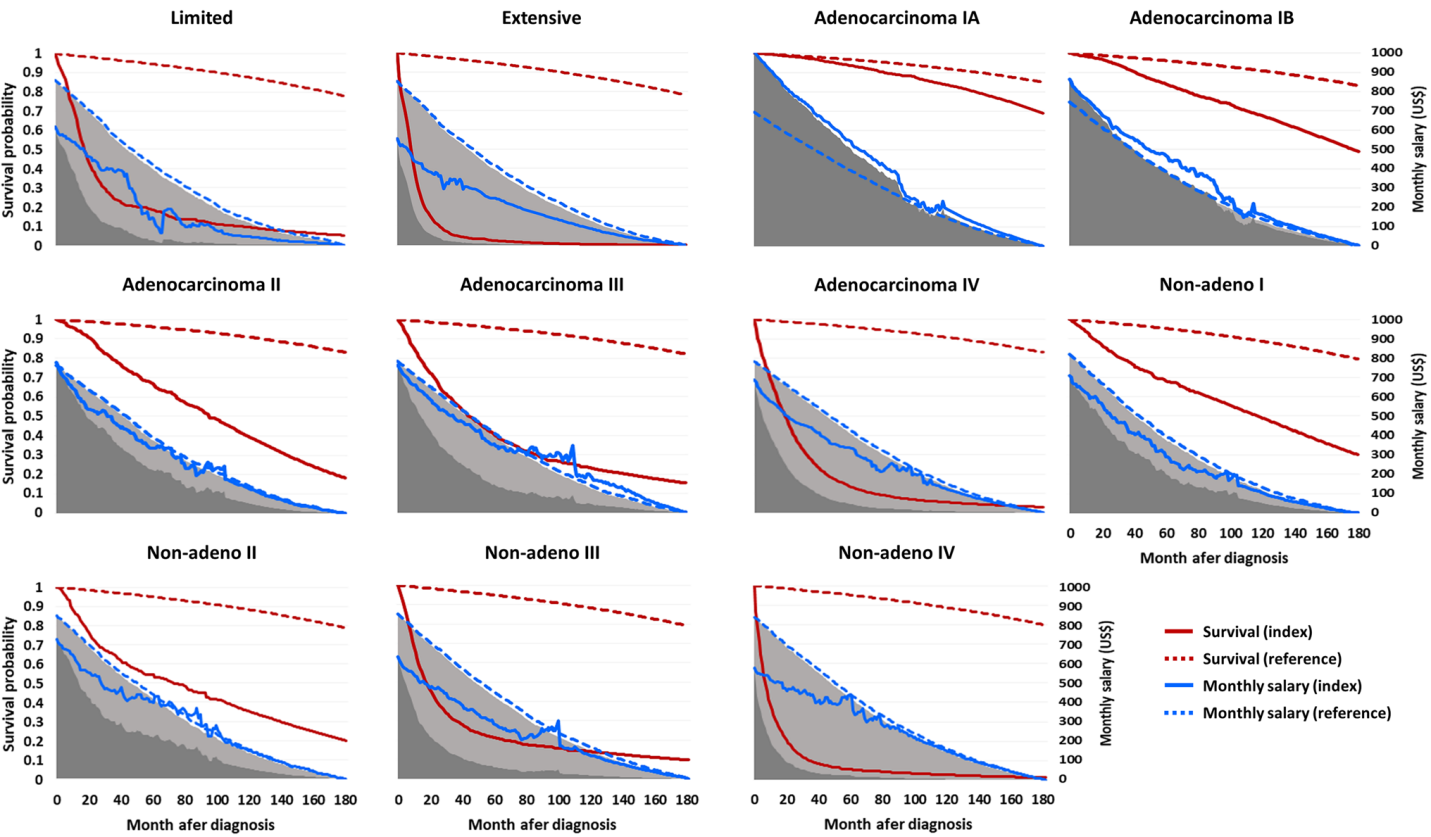
Lifetime productivities (dark shaded areas) and losses of lifetime productivity (light shaded areas) of lung cancer patients. The latter depict the differences in lifetime productivity between the index and age-, sex-, and calendar year-matched reference groups. The reddish curves represent the survival probabilities along time after diagnosis; the blue curves indicate the monthly working salaries; among them, the index groups were shown as solid lines, while the corresponding reference groups were expressed as dashed lines. Non-adeno: non-small cell lung cancers other than adenocarcinoma

Figure 1 displays the survival functions and EMRATIO curves of index patients and the age-, sex-, and calendar year-matched referents. The products of monthly survival probabilities and monthly EMRATIOs were summed to estimate the lifetime employment durations of the index and reference groups. Most lung cancer patients not only had shorter survival but also lower EMRATIOs compared to the referents, resulting in shorter lifetime employment durations. The difference in lifetime employment duration between the index and reference groups represented the loss of lifetime employment duration. Similarly, the products of monthly survival probabilities and monthly working salaries inferred from the NHI insurance premiums were summed to estimate the lifetime productivities of the index and reference groups (Fig. 2). The difference in lifetime productivity between the two groups indicated the loss of lifetime productivity.

Table 2 presents the lifetime employment duration and productivity of lung cancer patients and their corresponding referents for the losses of lifetime employment duration and productivity. Patients with SCLC or NSCLC other than adenocarcinoma experienced greater losses of lifetime employment duration and productivity as compared to adenocarcinoma patients. Compared to those diagnosed at an early stage, patients with advanced-stage lung cancer had greater losses of lifetime employment duration and productivity. For example, the average losses of lifetime employment duration and productivity for patients with stage IV adenocarcinoma were 2.6 years (74% of the corresponding referents’ duration) and US$40,531 (74% of the corresponding referents’ earnings), respectively. The average loss of lifetime employment duration for patients with stage IA adenocarcinoma was 0.1 years (3% of the corresponding referents’ duration), and the average loss of lifetime productivity was US$-17,970 (-32% of the corresponding referents’ earnings).

**Table 2:** Lifetime employment duration and productivity of lung cancer patients, compared with those of age-, sex-, and calendar year-matched referents

Pathology	Stage	LE, life-year (95% CI)	Lifetime employment duration, year (95% CI)	Loss of lifetime employment duration, year (95% CI)	Relative loss of lifetime employment duration, mean (95% CI)	Lifetime productivity, US$ (95% CI)	Loss of lifetime productivity, US$ (95% CI)	Relative loss of lifetime productivity, mean (95% CI)
SCLC	Limited	3.4 (2.8–5.0)	1.0 (0.9–1.1)	2.3 (2.1–2.5)	70% (60–81%)	12,886 (11,376–14,528)	42,375 (39,181–45,248)	77% (68–87%)
	Referents	23.7 (23.5–23.9)	3.3 (3.1–3.5)			55,261 (51,796–57,885)		
	Extensive	1.0 (1.0–3.5)	0.4 (0.4–0.5)	2.9 (2.7–2.9)	88% (79–91%)	5678 (5081–6321)	49,053 (47,073–50,709)	90% (84–96%)
	Referents	23.7 (23.5–23.9)	3.3 (3.2–3.4)			54,730 (52,871–56,353)		
Adeno	IA	19.2 (16.1–23.4)	3.6 (3.4–3.7)	0.1 (0.0–0.2)	3% (0–6%)	73,522 (68,901–77,259)	– 17,970 ( – 22,188 to ( – 13,935))	– 32% ( – 39 to ( – 26)%)
	Referents	26.7 (26.7–26.9)	3.7 (3.6–3.8)			55,552 (54,243–56,358)		
	IB	15.0 (12.7–18.2)	2.9 (2.7–3.1)	0.4 (0.2–0.6)	12% (6–19%)	52,291 (47,230–56,728)	– 1615 ( – 6096 to 3198)	– 3% ( – 12 to 7%)
	Referents	25.8 (25.6–26.0)	3.3 (3.2–3.4)			50,676 (49,151–52,838)		
	II	9.0 (8.3–15.6)	2.2 (1.9–2.5)	1.1 (0.8–1.3)	32% (23–41%)	35,682 (30,193–40,529)	16,246 (11,126–20,164)	31% (20–41%)
	Referents	25.7 (25.5–25.9)	3.4 (3.2–3.5)			51,928 (48,897–54,577)		
	III	6.9 (6.0–8.4)	1.8 (1.6–1.9)	1.7 (1.5–1.8)	50% (43–55%)	28,653 (25,281–31,262)	24,933 (21,301–27,920)	47% (38–54%)
	Referents	25.4 (25.3–25.6)	3.4 (3.3–3.5)			53,586 (51,910–55,497)		
	IV	2.8 (2.7–4.4)	1.0 (0.9–1.0)	2.6 (2.5–2.6)	74% (69–74%)	14,211 (13,587–14,875)	40,531 (39,757–41,448)	74% (72–77%)
	Referents	25.7 (25.7–25.9)	3.5 (3.5–3.6)			54,742 (54,063–55,358)		
Non-adeno	I	10.9 (9.2–13.6)	2.3 (2.1–2.6)	0.9 (0.6–1.2)	27% (18–39%)	33,311 (28,450–38,592)	19,420 (14,829–23,795)	37% (27–48%)
	Referents	24.3 (24.1–24.6)	3.3 (3.1–3.4)			52,731 (49,807–55,535)		
	II	8.4 (7.0–13.0)	2.2 (1.9–2.6)	1.2 (0.9–1.4)	35% (25–44%)	32,383 (26,596–39,666)	24,090 (17,893–29,491)	43% (30–56%)
	Referents	24.1 (23.8–24.4)	3.4 (3.2–3.6)			56,473 (52,776–60,221)		
	III	4.4 (3.7–5.8)	1.2 (1.0–1.3)	2.3 (2.2–2.5)	66% (61–74%)	15,270 (13,616–17,081)	42,241 (39,774–44,893)	73% (67–81%)
	Referents	24.2 (24.1–24.4)	3.5 (3.4–3.6)			57,511 (55,362–59,567)		
	IV	1.4 (1.4–3.0)	0.6 (0.5–0.6)	2.9 (2.8–3.0)	83% (80–88%)	7470 (6784–8249)	49,407 (47,856–50,813)	87% (82–92%)
	Referents	24.5 (24.4–24.6)	3.5 (3.4–3.5)			56,877 (55,285–58,099)		

*CI* confidence interval, *LE* life expectancy, *Non-adeno* non-small cell lung cancers other than adenocarcinoma, *SCLC* small cell lung cancer

### Savings of lifetime employment duration and productivity

The losses of lifetime employment duration and productivity were smaller for lung cancer patients who were never smokers as compared to all lung cancer patients (Table 3). We applied the estimates to 100 lung cancer patients with a shift in pathology and stage distribution based on the first-round screening of TALENT. The savings of lifetime employment duration were 132.2 (95% prediction interval: 116.2–147.4) years, and the savings of lifetime productivity were 3353 (95% prediction interval: 2914–3802) thousand US dollars, or around 118.5 per capita gross domestic product in 2020.

**Table 3:**
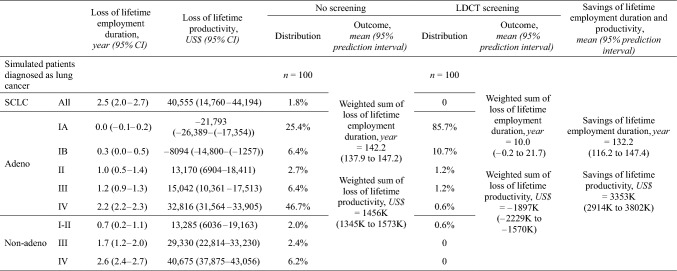
Multiplying losses of lifetime employment duration and productivity by the shift in pathology and stage distribution based on the first-round screening of TALENT to estimate savings of lifetime employment duration and productivity

*CI* confidence interval, *LDCT* low-dose computed tomography, *Non-adeno* non-small cell lung cancers other than adenocarcinoma, *SCLC* small cell lung cancer, *TALENT* Taiwan Lung Cancer Screening in Never Smoker Trial

## Discussion

In addition to mortality-associated loss of productivity, this study also considered the productivity loss related to lung cancer morbidity. We used real-world data to depict the dynamic changes in the employment status and working salaries of lung cancer survivors and their matched referents (Figs. 1 and 2). Although the loss of lifetime productivity in lung cancer patients mainly resulted from the shorter survival and loss of LE, lower employment rates and working salaries also played a role in increasing the loss of productivity. Furthermore, the losses of lifetime employment duration and productivity for patients with advanced-stage lung cancer were substantially greater than those for early-stage patients (Table 2), highlighting the importance of early detection. Implementing LDCT screening in high-risk populations could not only save lives but also extend employment duration and preserve lifetime productivity (Table 3).

To estimate lifetime productivities, we used monthly working salaries inferred from NHI insurance premiums instead of relying on national average incomes that varied by age, sex, and calendar year [11]. Lung cancer patients had a higher likelihood of temporary or permanent dropout from the job market compared to the general population [7, 8, 23]. Due to physical and psychological barriers, lung cancer patients would also be prone to a shift from full-time to part-time job after diagnosis. Instead of applying the same national average incomes to both groups, we used working salaries calculated from insurance premiums to capture the reduced productivity while on the job (presenteeism). The advantage of this method was supported by the higher percentages of relative loss of lifetime productivity than the relative loss of employment duration in patients with SCLC and NSCLC other than adenocarcinoma (Table 2). To the best of our knowledge, this study may be the first to apply inferred salaries based on insurance premiums to quantify the productivity loss for cancer survivors.

Patients with SCLC, stage III/IV NSCLC had lower monthly EMRATIOs and working salaries than their matched referents right after diagnosis (Figs. 1 and 2). These findings could be attributed to the rapid tumor progression and treatment-related adverse events of advanced-stage patients, forcing them to leave or quit their jobs. Additionally, most patients with advanced-stage lung cancer received care at tertiary referral centers without evening or night services. Patients are usually required to take leave from work for doctor visits, forcing them to change to less burdensome jobs. In contrast, the monthly EMRATIOs of patients with stage I NSCLC and their referents did not differ a lot. In fact, patients with stage IA adenocarcinoma had higher monthly working salaries than their referents, resulting in higher lifetime productivity. A plausible explanation is that this subgroup of patients was more likely to be wealthy and paid out-of-pocket expenses for LDCT screening to detect the cancer early. Those with higher earnings might face greater workplace stress and have a higher probability of being ill [24]. This conjecture is supported by the higher monthly insurance premiums of patients with stage IA adenocarcinoma compared with the others (Table 1).

Different methods were used to value productivity [25]. The friction-cost method considers the productivity loss due to temporary absenteeism from an employer’s perspective. The human capital method calculates the loss of a worker’s lifetime earnings due to morbidity or premature death from a patient’s perspective. This study used the latter approach and extrapolated the estimates beyond the observable time horizon to account for lifetime earnings and societal impact of LDCT screening. As a result, the estimates obtained were greater than those using the friction-cost method.

Several limitations must be acknowledged in our study. First, we used monthly working salaries to calculate productivity loss and did not consider income from property rentals and firm profits, which might underestimate lifetime productivities of the index and reference groups. However, a 2.11% supplementary premium for receiving other types of income was charged and the NHI enforced the stipulation [26]. The magnitude of underestimation would not be too large. Additionally, an increase in life expectancy leads to increased consumption, which in turn improves welfare [27–29]. Our estimation did not concern loss of consumption associated with loss of life expectancy. The loss of lifetime productivity would be underestimated. Second, the National Cancer Registry data included smoking information on lung cancer patients beginning from 2011. They were followed up until the end of 2020 for monthly employment status and working salaries. Given the 10-year follow-up period, we needed to extrapolate the EMRATIOs of the index patients aged 50–59 years up to age 65. Nevertheless, a follow-up of 10 years is generally longer than the LE of lung cancer patients (Table 2), implying a relatively small bias might have existed. Third, our results cannot be generalized to populations outside of Taiwan since the survival, employment rates, and working salaries among different countries are supposed to be different. However, this methodology provides an alternative way for estimating the productivity loss of cancer survivors or patients with other chronic illnesses.

## Conclusion

In conclusion, this study used real-world EMRATIOs and monthly salaries to quantify the losses of lifetime employment duration and productivity in patients with different subtypes and stages of lung cancer. Compared with adenocarcinoma patients, those with SCLC or NSCLC other than adenocarcinoma experienced greater losses of lifetime employment duration and productivity. Early diagnosis of lung cancer would save losses of employment duration and lifetime productivity for patients. Future evaluation of the cost-effectiveness of lung cancer screening could consider including these societal impacts.

### Supplementary Information

Below is the link to the electronic supplementary material.

Supplementary file1 (DOC 141 KB)

## Data Availability

Data are available from the corresponding author conditional on permission from the involved committee and national register.
