# Supplementary material for: Losses of lifetime employment duration and productivity for patients with different subtypes and stages of lung cancer
Source: Eur J Health Econ. 2023 Aug 7;25(5):775–85. doi: 10.1007/s10198-023-01624-4 (PMC11192829; doi:10.1007/s10198-023-01624-4)
Supplement: Supplementary file 1 — Supplementary file1 (DOC 141 KB) [file 10198_2023_1624_MOESM1_ESM.doc]

Supplementary information

**Losses of lifetime employment duration and productivity for patients with different subtypes and stages of lung cancer**

Running title: Loss of employment and productivity for lung cancer

**Supplementary Table 1.** Validation of the extrapolated estimates

|  | | Number | Estimates using the extrapolation based on the first 5 years of follow-up | 10-year follow-up Kaplan-Meier estimate | Relative bias |
| --- | --- | --- | --- | --- | --- |
|  |  |  | *mean (SE) month* | *mean (SE) month* | *%* |
| SCLC | Limited | 379 | 33.9 (2.3) | 32.1 (1.8) | 5.7 |
|  | Extensive | 951 | 12.9 (0.7) | 13.1 (0.6) | -1.6 |
| Adeno | IA | 2274 | 108.9 (1.7) | 110.2 (0.5) | -1.2 |
|  | IB | 1039 | 97.6 (2.0) | 100.1 (1.1) | -2.5 |
|  | II | 496 | 79.6 (2.7) | 80.4 (1.9) | -1.0 |
|  | III | 1289 | 54.8 (1.4) | 56.9 (1.2) | -3.6 |
|  | IV | 7508 | 26.6 (0.3) | 27.7 (0.3) | -4.1 |
| Non-adeno | I | 396 | 83.0 (2.8) | 85.4 (2.2) | -2.8 |
|  | II | 284 | 70.0 (3.5) | 69.8 (2.8) | 0.2 |
|  | III | 1092 | 35.7 (1.4) | 36.7 (1.2) | -2.9 |
|  | IV | 1897 | 13.7 (0.6) | 14.6 (0.5) | -6.4 |

Non-adeno: non-small cell lung cancers other than adenocarcinoma; SCLC: small cell lung cancer; SE: standard error.
